# Correction to ‘Post-fire spectral recovery and driving factors across the boreal and temperate forests’ (2025), by Kai *et al.*

**DOI:** 10.1098/rstb.2025.0256

**Published:** 2025-09-04

**Authors:** Kaili Li, Zhihua Liu, Wenru Xu, Wenjuan Wang, Jiajia Su, Qiushuang Lv, Wenhua Guo, Marie Johnson

**Affiliations:** ^1^ CAS IAE, Shenyang, People’s Republic of China

**Keywords:** forest fire, spectral forest recovery, spectralindices, fire severity, climatic factors

*Phil. Trans. R. Soc. B*, **380,** 20230453 (Published online 17 April 2025). (https://doi.org/10.1098/rstb.2023.0453).

An author’s name was incorrectly spelt in the original publication. The first author was listed as Li Kai Li in the original version, and this has been corrected to Kaili Li.

